# Structural correlates of affinity in fetal versus adult endplate nicotinic receptors

**DOI:** 10.1038/ncomms11352

**Published:** 2016-04-22

**Authors:** Tapan Kumar Nayak, Srirupa Chakraborty, Wenjun Zheng, Anthony Auerbach

**Affiliations:** 1 Department of Physiology and Biophysics, State University of New York at Buffalo, Buffalo, New York 14214, USA; 2 Department of Physics, State University of New York at Buffalo, Buffalo, New York 14260, USA

## Abstract

Adult-type nicotinic acetylcholine receptors (AChRs) mediate signalling at mature neuromuscular junctions and fetal-type AChRs are necessary for proper synapse development. Each AChR has two neurotransmitter binding sites located at the interface of a principal and a complementary subunit. Although all agonist binding sites have the same core of five aromatic amino acids, the fetal site has ∼30-fold higher affinity for the neurotransmitter ACh. Here we use molecular dynamics simulations of adult versus fetal homology models to identify complementary-subunit residues near the core that influence affinity, and use single-channel electrophysiology to corroborate the results. Four residues in combination determine adult versus fetal affinity. Simulations suggest that at lower-affinity sites, one of these unsettles the core directly and the others (in loop E) increase backbone flexibility to unlock a key, complementary tryptophan from the core. Swapping only four amino acids is necessary and sufficient to exchange function between adult and fetal AChRs.

## Abstract (web summary)

Adult and fetal nicotinic acetylcholine receptors (AChRs) have different functional requirements and affinity for ACh. Here, the authors use molecular dynamics and electrophysiology to investigate this affinity, and identify four amino acids that when swapped exchange function between adult and fetal AChRs.

## Graphical abstract

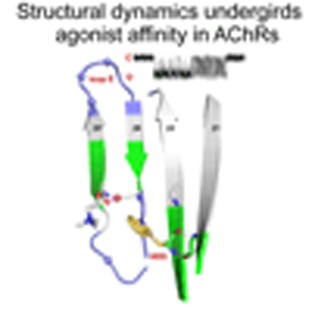

Endplate AChRs are heteropentamers that have two α(1) subunits and one each of β, δ and either γ or ɛ. Each receptor has two functional neurotransmitter binding sites located in the extracellular domain at subunit interfaces, either αδ+αγ (fetal) or αδ+αɛ (adult) (Fig. 1a, inset). Agonist affinities in mouse AChRs have been measured for these sites, both separately and as pairs^1^,^2^,^3^,^4^. The resting equilibrium dissociation constant for ACh (*K*_d_^ACh^) is ∼30-fold lower at αγ compared with αδ or αɛ. The affinity of the fetal, αγ-site is similar to that of the *Lymnaea stagnalis* acetylcholine binding protein (AChBP)^5^.

The fetal, γ-subunit is required for the proper maturation of the neuromuscular synapse^6^,^7^,^8^. In mice, γ-null mutations are lethal^9^ and in humans γ-subunit mutations cause both lethal and non-lethal (Escobar) types of multiple pterygium syndromes^10^,^11^,^12^. The reason(s) for the γ-subunit requirement in synaptogenesis is not known, but possibilities include the higher agonist affinity, smaller single-channel conductance, longer open-channel lifetime, smaller gating-voltage dependence, lower Ca^++^ permeability and lower probability of opening constitutively of fetal AChRs^1^,^13^,^14^. Physiologically, the higher affinity of the fetal agonist binding site for both ACh and choline will lead to larger cellular responses^1^,^15^ at the low concentrations of these agonists that prevail at developing neuromuscular synapses^16^.

There are ∼250 side chain differences between the adult ɛ- and fetal γ-subunits. Mutational correlates of differences between the fetal and adult AChR function have been investigated previously. The mice expressing AChRs having an ɛ–γ-subunit chimera joined at a conserved glycine in loop E, γ(≤113)+ɛ(≥114), have a fetal-like open-channel lifetime and adult-like conductance^7^. Synapses expressing these AChRs undergo normal endplate differentiation but show altered innervation patterns. In mouse AChRs, the M3–M4 linker has been shown to influence fetal versus adult open-channel lifetime^17^. In *Xenopus* AChRs, residues at positions 6′/7′ in M2 (N/I in γ versus S/V in ɛ) were implicated in setting fetal versus adult conductance^18^, and in rat AChRs, swapping the M2–20′ amino acid from K to Q results in a partial exchange of conductance^19^. None of the above studies investigated the structural basis of fetal versus adult affinity, which is the topic we address here.

At all three kinds of agonist site (αδ, αɛ and αγ), ACh is stabilized in the binding pocket by a core of five aromatic residues (Fig. 1a). On the basis of their individual contributions to affinity, these can be divided into two working groups, an ‘aromatic triad' and a ‘special pair'^20^. The triad is αW149 (indole ring), αY198 (benzene ring) and αY190 (benzene ring and hydroxyl). The four functional groups of these three amino acids each make a similar contribution to ACh binding energy at all the three agonist sites^15^,^21^. In adult AChRs, these groups provide almost all of the neurotransmitter binding energy, which is equal to +0.59 ln*K*_d_ (23 °C). Together, these groups stabilize ACh by approximately −5.1 kcal M^−1^ per site (−10.2 kcal M^−1^ for αδ+αɛ sites combined). The action of the triad is led by αY190, because the ring and –OH each contribute approximately −2 kcal M^−1^ at αδ, αɛ and αγ.

A second working part of the agonist site apparatus is the special pair, αY93 (benzene ring) and ɛ/γW55 (δW57; indole ring). At αɛ and αδ, these two groups make only a small contribution to ACh affinity, but at the fetal αγ site, the aromatic rings contribute significantly to bring the single-site-total to approximately −7.1 kcal M^−1^ and the αδ+αγ total to −12.2 kcal M^−1^ (ref. 15). Moreover, at αγ, the effects of alanine substitutions of this pair are not independent, as removing either ring interferes substantially with the other's ability to stabilize ACh. The energy contribution of this tryptophan differs massively between the fetal and adult sites. At αγ, the γW55A mutation decreases affinity by ∼2,000-fold, whereas the homologous substitution at αɛ reduces it by only ∼13-fold and at αδ it actually increases affinity by ∼2-fold.

Because the aromatic core has the same composition at all the three kinds of agonist sites, the difference in affinity between the fetal and adult AChRs can be attributed to residues in the complementary, non-α-subunit (δ, ɛ or γ). Our approach to finding these amino acids was to use molecular dynamics (MD) simulations of homology models based on AChBP to identify complementary-subunit amino acids near the core that influence the contribution of the special pair to affinity. Then, we exchanged those side chains *in vitro* (δ and ɛ↔γ) and estimated affinity from single-channel currents of AChRs expressed in cells. The results indicate that four residues in combination determine adult versus fetal resting affinity, three in loop E (111–113; γ-subunit numbers) and one in the β5–β5′ linker (104). In the β5′–β6 hairpin region, mutations of the human γ-subunit (V108, S111, P112 and P121) cause multiple pterygium and Escobar syndromes^12^. The simulations suggest that these amino acids influence core properties by changing the structure and dynamics of the β5–β5′ linker and the complementary β-sheet, to effect the action of the special pair and the affinity for the agonist. Swapping the four side chains is both necessary and sufficient to exchange fetal versus adult affinities and open-channel lifetimes.

The results are presented in five sections. First, we build and test homology models for the fetal and adult agonist sites. Second, we use the models to identify residues in the complementary subunit that influence ACh affinity *in silico*. Third, we use electrophysiology to measure *in vitro* affinities of AChRs having the identified residues swapped, fetal↔adult. Fourth, we report the effects of point mutations on affinity, from the identified group and of a critical, complementary-side tryptophan. Fifth, we analyse the simulated structures and develop hypotheses for the mechanisms that undergird the fetal versus adult affinity difference.

## Results

### Tuning the model

**Figure 1:**
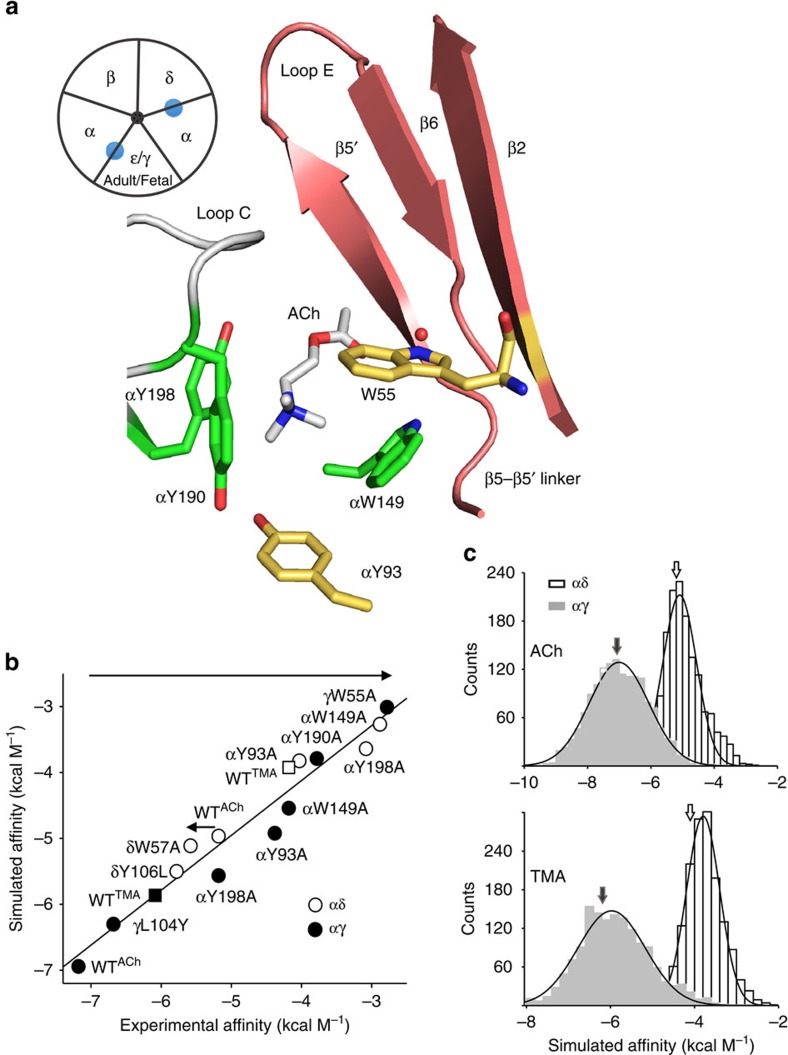
Ligand binding site and affinities. (**a**) The principal subunit (α in AChRs) is left (white) and the complementary subunit (δ, ɛ or γ) is right (pink; *Lymnaea stagnalis*, pdb accession number 3WIP^27^). Aromatic triad, green; special pair, yellow; structural water, red sphere. Inset: in endplate AChRs only two of the five subunit interfaces are functional binding sites (blue). (**b**) Affinity estimates from simulations and electrophysiology experiments are correlated. Circles, ACh (alanine mutations of core aromatics except where marked); squares, TMA (WT only). In both simulations and electrophysiology experiments, there is a large loss in binding energy with γW55A and a small gain with δW57A (arrows). (**c**) Distributions of binding energies from WT AChR simulations. Arrows mark the corresponding *in vitro* affinities estimated from electrophysiology experiments.

Our approach was to use MD simulations to guide mutagenesis, and electrophysiology experiments to measure *in vitro* affinities. We used an empirical and computationally inexpensive method of estimating agonist binding energy *in silico* (see Methods section). By adjusting the free parameters for van der Waals and electrostatic contributions in the calculations, simulated and experimental affinities could be correlated (Fig. 1b). This was possible, in part, because in AChRs the entropy component of affinity is small^22^,^23^.

The circles in Fig. 1b are simulated versus experimental ACh binding energies for αγ and αδ agonist sites with mutations of core residues. The free parameters in the binding energy calculation (Equation 1, Methods section) were obtained only from the alanine mutations. Two non-alanine substitutions fell on the same regression line. The arrows indicate that the large and opposite effect of an alanine substitution of the complementary tryptophan on affinity at αγ versus αδ that is apparent *in vitro* was reproduced *in silico*. Also, in both simulations and experiments the αγ^WT^ site showed ∼40% more favourable binding energy for ACh and tetramethylammonium (TMA) compared with the αδ^WT^ site (Fig. 1c). These results suggest that brief simulations and approximate binding energy estimates can be used to screen for amino acids that influence affinity.

### C4 in silico

**Figure 2:**
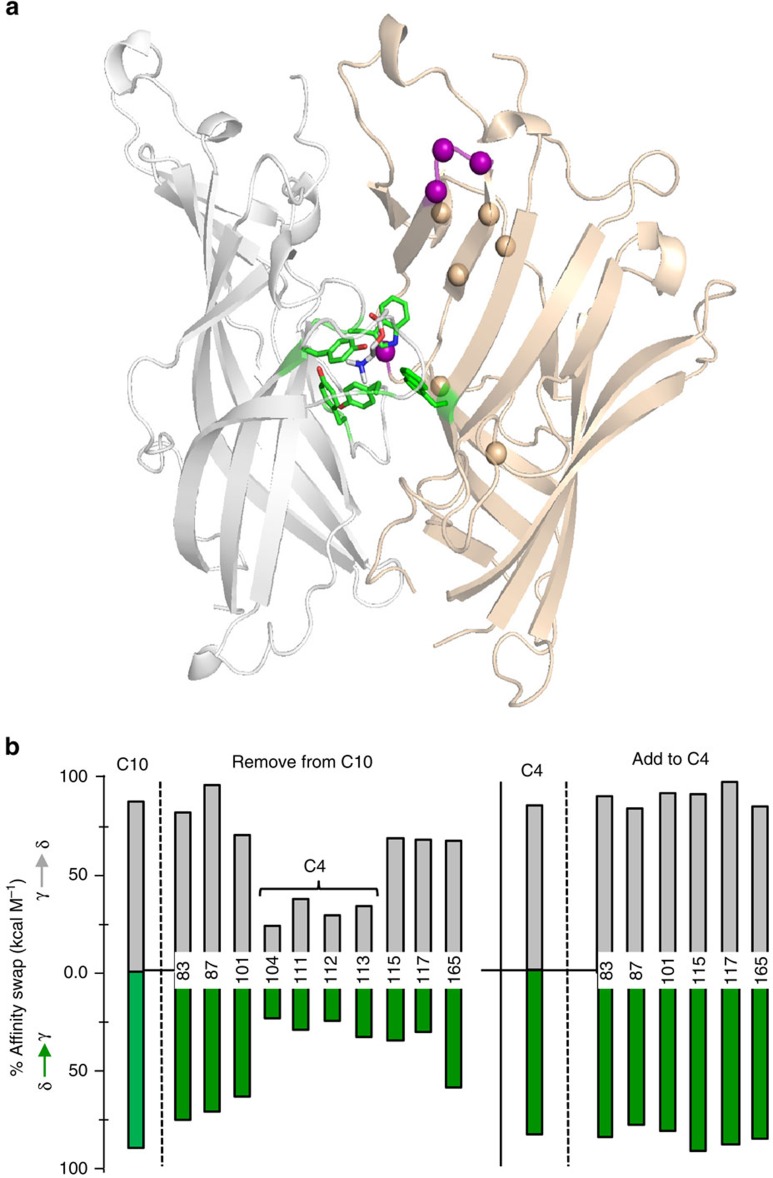
Simulations identify affinity-influencing side chains. (**a**) Ten residues in the complementary subunit (tan) are within 20 Å of the ACh quaternary amine and are different in γ versus δ≈ɛ (large spheres; C10). Core, green; C4 residues, violet. (**b**) Affinity changes calculated *in silico* for different combinations of side-chain swaps. Residue numbers pertain to the γ-subunit. Left-to-right: swapping all C10 residues almost completely exchanges affinity; dropping any one of four residues from C10 reduces the magnitude of the affinity change (C4; violet spheres in **a**); swapping all C4 side chains together is sufficient to exchange affinity; adding residues does not improve significantly the effect of the C4 swap.

The first goal was to identify residues in the complementary, γ- versus δ-subunit that are responsible for the 30-fold higher affinity for ACh in αγ versus αδ. We used two criteria to select candidate residues to be exchanged *in silico*. We chose side chains that in the model were within 20 Å of the quaternary ammonium (QA) group of ACh and were different between γ and δ but homologous between ɛ and δ. A total of 10 amino acids satisfied both of these criteria (C10; Fig. 2a).

In the first set of simulations, all the C10 side chains were swapped, γ→δ and δ→γ, and ACh binding energies were calculated. The results were that the C10-mutated αδ site provided about the same binding energy as the αγ^WT^ site, and the C10-mutated αγ site provided about the same as the αδ^WT^ site (Fig. 2b). This suggested that the mutation(s) we were seeking were within the C10 group.

We then winnowed the mutation list. First, each member of the C10 group was dropped one at a time. The result of these nine-swap simulations was that the affinities no longer reversed when one of 4 of the original 10 amino acids was omitted. When γ(L104, S111, P112 or D113) or δ(Y106, Y113, D114 or S115) side chains remained as in the parent subunit, the full affinity reversal observed for C10 was incomplete. We call this set of four complementary subunit side chains C4.

Next, we simulated affinities using models in which only the C4 amino acids were exchanged as a group, to make constructs αγ^C4δ^ and αδ^C4γ^ (regular text is parent and superscript is target). Swapping all C4 side chains was sufficient to make the αγ site have an affinity like αδ^WT^ and the αδ site to have an affinity like αγ^WT^. *In silico*, exchanging the C4 set of side chains was sufficient to exchange affinities, stably (Fig. 2b).

In the next set of simulations, each of the six other residues of the C10 starting set was added to C4, one at a time. None of these made a significant difference in the magnitude of the affinity swap. Finally, we dropped each mutation from the C4 set, one at a time, and estimated ACh affinity. Unlike in the above simulations, the trajectories of these three-mutant swaps were not stable (both fluctuating and divergent root mean square deviation; Supplementary Fig. 1). *In silico*, C4 was the minimum construct required for exchanging stably αγ- and αδ-binding energies.

### C4 *in vitro*

**Figure 3:**
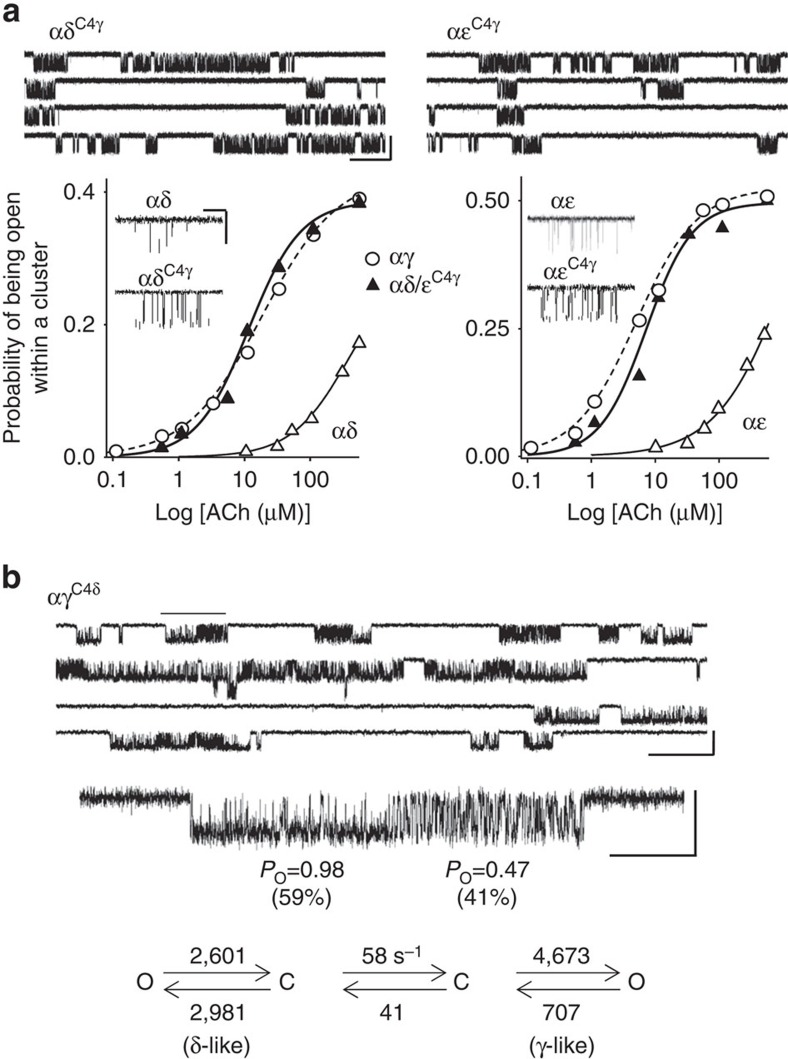
Electrophysiology measurements corroborate simulations. (**a**) Replacing all of the δ/ɛ C4 side chains with those of γ (αδ/ɛ^C4γ^) fully exchanges ACh affinity. In all traces, openings are down. Top, low time-resolution view of single-channel currents (scale bar: vertical, 4 pA; horizontal, 0.5 s). The long gaps between clusters of openings are sojourns in desensitized states; affinity was estimated from the intra-cluster interval durations; membrane potential was +70 mV to reduce channel block by ACh (100 mM); only one agonist site was functional. Bottom, dose-response curves. Inset, example clusters (10 μM ACh, *V*_m_=−100 mV, no channel block, scale bar: vertical, 7 pA; horizontal, 0.1 s). (**b**) Top, replacing all of the γ C4 side chains with those of δ (αγ^C4δ^) generates AChRs that switch between fetal and adult affinity and open-channel lifetime (100 mM ACh, *V*_m_=+70 mV, scale bar: vertical, 4 pA; horizontal, 0.5 s). Bottom, bimodal kinetics of single-channel intra-cluster interval duration (scale bar: vertical, 4 pA; horizontal, 50 ms) showing the αγ↔αδ switch in behaviour.

We next made corresponding experimental measurements of ACh affinity (*K*_d_) from single-channel currents recorded from AChRs expressed in HEK cells (Fig. 3). The C4 residues were investigated at αγ-, αδ- and αɛ-binding interfaces. In these electrophysiology experiments, the constructs were αδ^C4γ^ (δY106L+δY113S+δD114P+δS115D), αɛ^C4γ^ (ɛY104L+ɛY111S+ɛE112P+ɛG113D) and αγ^C4δ^ (γL104Y+γS111Y+γP112D+γD113S).

First, the *in vitro* measurements were made using AChRs that had only one functional binding site, its companion being knocked out by a mutation(s)^24^. The results were that the C4 ɛ→γ and δ→γ exchanges resulted in a nearly complete swap of affinity (Fig. 3a). The αɛ^C4γ^ and αδ^C4γ^ constructs each had the dose-response profile and affinity of the target, αγ^WT^ site. The *K*_d_^ACh^ values for αɛ^C4γ^ and αδ^C4γ^ are shown in Supplementary Table 1.

The behaviour of αγ^C4δ^ was more complex (Fig. 3b). This construct alternated between two distinct modes of single-channel activity. One mode retained the high-affinity (Supplementary Table 1) and longer open-channel lifetime characteristic of the parent (αγ^WT^) and the other had the low-affinity and briefer open-channel lifetime characteristic of the target (αδ^WT^). The prevalence of each mode was approximately equal (isoenergetic), with a switching time constant of ∼20 ms. The C4 γ→δ affinity exchange *in silico* was stable, but *in vitro* the swap in binding and gating functions was bimodal.

**Figure 4:**
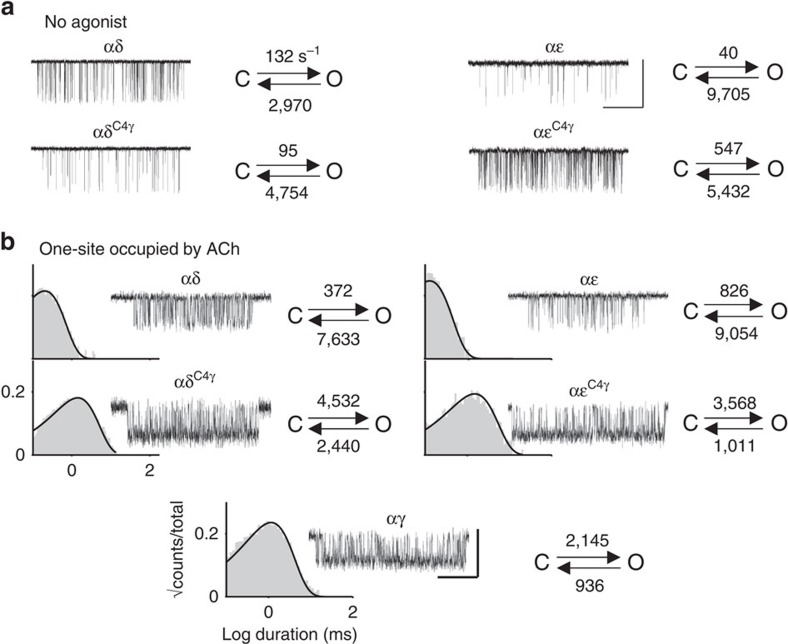
C4 mutations swap fetal versus adult gating kinetics. (**a**) Gating without agonists (openings are down; *V*_m_=−100 mV, scale bar: vertical, 7 pA; horizontal, 250 ms). At αδ and αɛ, the effects of the C4γ mutations are <2-fold except for the αɛ opening rate constant. C4 mutations have a small effect on unliganded gating. (**b**) Gating with one functional agonist binding site occupied by ACh (100 mM; *V*_m_=+70 mV to reduce channel block, scale bar: vertical, 4 pA; horizontal, 50 ms). At αδ and αɛ, the C4γ mutations cause the channel-closing rate constant to become like that of the target subunit. Histograms are open-channel lifetime distributions. See Fig. 5a for summary.

**Figure 5:**
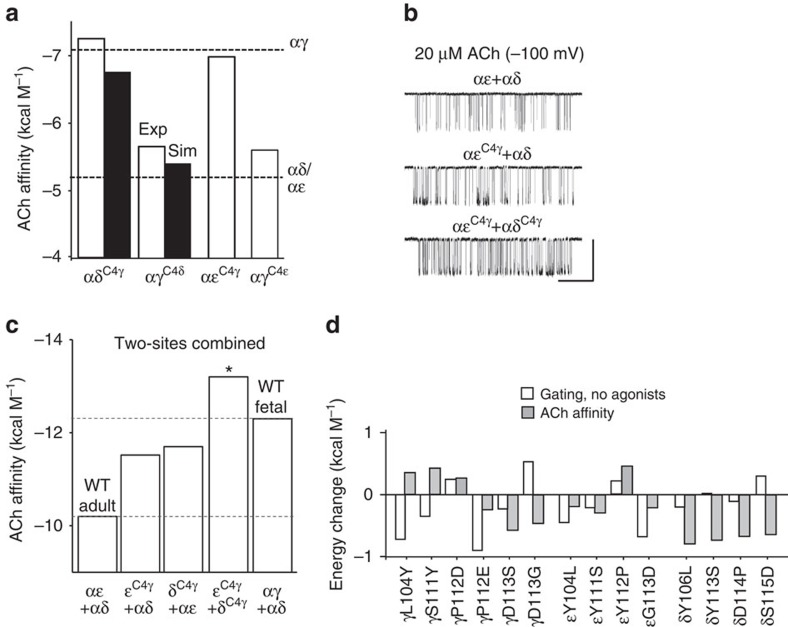
ACh affinities for one- and two-site AChRs. (**a**) ACh affinities (+0.59 lnK_d_) of C4-swapped constructs in AChRs having a single functional agonist binding site (exp, from electrophysiology; sim, from MD simulations; see Supplementary Table 1). Dashed lines, WT affinities measured by using electrophysiology^15^. The C4 mutations exchange affinity. (**b**) Swapping C4 at both adult sites (αɛ+αδ) in two-site AChRs. Example single-channel currents (openings are down, scale bar: vertical, 7 pA; horizontal, 50 ms). (**c**) Total ACh binding energy from both sites combined; *, the doubly swapped adult AChR (ɛ^C4γ^+δ^C4γ^) has even-more favourable ACh binding energy than the WT fetal AChR. (**d**) Effects of swapping C4 side chains one at a time in one-site receptors.

In addition to decreasing the ACh equilibrium dissociation constant (increasing affinity), the αɛ/δ^C4γ^ mutations generated a fetal-like, slower channel-closing rate constant in the presence of the neurotransmitter ACh. The effects of these C4γ mutations in the absence of agonists or in the presence of saturating [ACh] are shown in Fig. 4a,b, with the results summarized in Fig. 5a and Supplementary Table 1. The C4 substitutions were effective in exchanging ACh affinity. We also measured affinity for the partial agonists carbamylcholine (CCh), TMA and choline and these exchanged affinity partially with C4 mutations. On average, the low→high affinity C4 swaps were ∼90% effective and the high→low affinity swaps were ∼70% effective.

We also made *in vitro* measurements from AChRs having only three of the C4 side-chain exchanges. The kinetic properties of these C3 receptors were complex and we did not attempt to estimate affinity.

The two WT AChR agonist sites act nearly independently in so far as binding energies measured from two sites combined are approximately the sums of single-site energies^15^. In the next set of experiments, we examined *in vitro* two different mutated, adult AChRs (both sites functional), αɛ^C4γ^+αδ^WT^ and αɛ^C4γ^+αδ^C4γ^. The prediction was that the first would have a fetal-type affinity and the second would be a ‘super' AChR having the high, fetal-type affinity at both agonist sites. These expectations were confirmed, approximately (Fig. 5b,c).

### Point mutations

The effects of point mutations of each C4 residue at each agonist site are shown in Fig. 5d. In experiments, none of these single exchanges had a large (>1 kcal M^−1^) effect on affinity or gating without agonists. With regard to affinity, the sum of the δ→γ single-residue C4 swaps was −2.8 kcal M^−1^, which is close to that for the αδ^C4γ^ combination, but the sum of the γ→δ single-residue swaps was only ∼+0.5 kcal M^−1^. With regard to the allosteric constant, none of the C4 swap sums were sufficient to account for the ∼14-fold smaller unliganded gating equilibrium constant (+1.6 kcal M^−1^) apparent in fetal versus adult AChRs at −100 mV (ref. 1).

The mouse AChR ɛ-γ chimera was joined at a conserved glycine in loop E^7^. We were unable to record single-channel currents from AChRs having a point substitution here (γG114 to A, L or P) because these receptors either failed to express or open.

**Figure 6:**
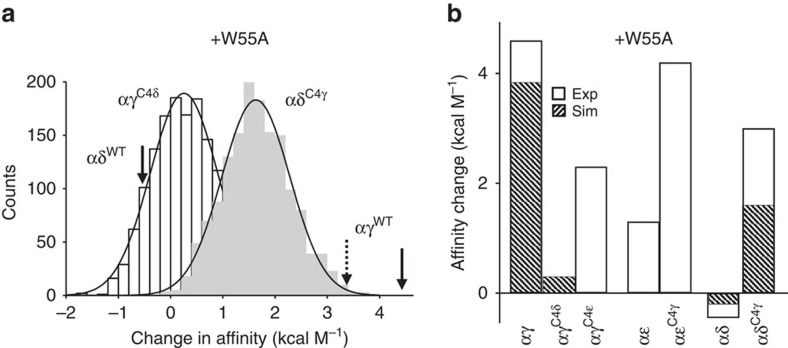
The action of W55 in C4-swapped constructs. (**a**) Change in ACh affinity with an added W55A mutation. Histograms simulated from C4-swapped constructs; solid arrows, electrophysiology results in WT background (see Fig. 1b). Left, for the αγ^C4δ^ and αδ^WT^ backgrounds, the change in affinity was similar and small in both simulations and experiments. Right, for the αδ^C4γ^ and αγ^WT^ backgrounds, the simulated change in affinity was smaller than observed in experiments. Dashed line, simulated change in affinity for the αγ^WT^ background. (**b**) Summary of W55A mutational effects on ACh affinity. In both the simulations and experiments, the energy change was partial for αγ^C4δ^ and αδ^C4γ^ but nearly complete for αɛ^C4γ^.

The action of the complementary, special pair tryptophan is the main distinction between αγ versus αδ sites^15^,^25^,^26^. The huge difference in the effect of an alanine substitution at W55 in γ versus δ *in vitro* was also apparent *in silico* (Fig. 1b). To further test both the model and the ability of C4 to swap the properties of the aromatic core, we compared simulated and experimental ACh binding energies for the C4 constructs in the presence of the point mutation γ/ɛW55A or δW57A. If C4 was completely responsible for the different contributions of the special pair, we expected that an alanine substitution would have either no or little effect at αγ^C4δ/ɛ^ (as in αδ/ɛ^WT^), but would cause a large decrease in affinity at αδ/ɛ^C4γ^ (as in αγ^WT^). The results agreed with these predictions, but partially (Fig. 6). We did not test αγ^C4δ^(+δW55A) in electrophysiology experiments because αγ^C4δ^ gave rise to complicated, heterogeneous responses without the tryptophan mutation (Fig. 3).

### Analyses of structures

The modelling and electrophysiology results suggest that the β-sheet of the complementary surface of the agonist site can fold stably into, or isomerize between (Fig. 3b), either of two approximately isoenergetic configurations to produce a high, γ-like or a low, δ-like resting affinity (HA or LA, not to be confused with active-state vs resting-state affinities). Further, they suggest that the C4 side chains bias which of these super-secondary structures is adopted. Because there are no atomic-resolution structures of fetal or adult AChR agonist sites with agonist bound, we analysed structures generated in the MD simulations to explore possible difference between the alternate conformations.

**Figure 7:**
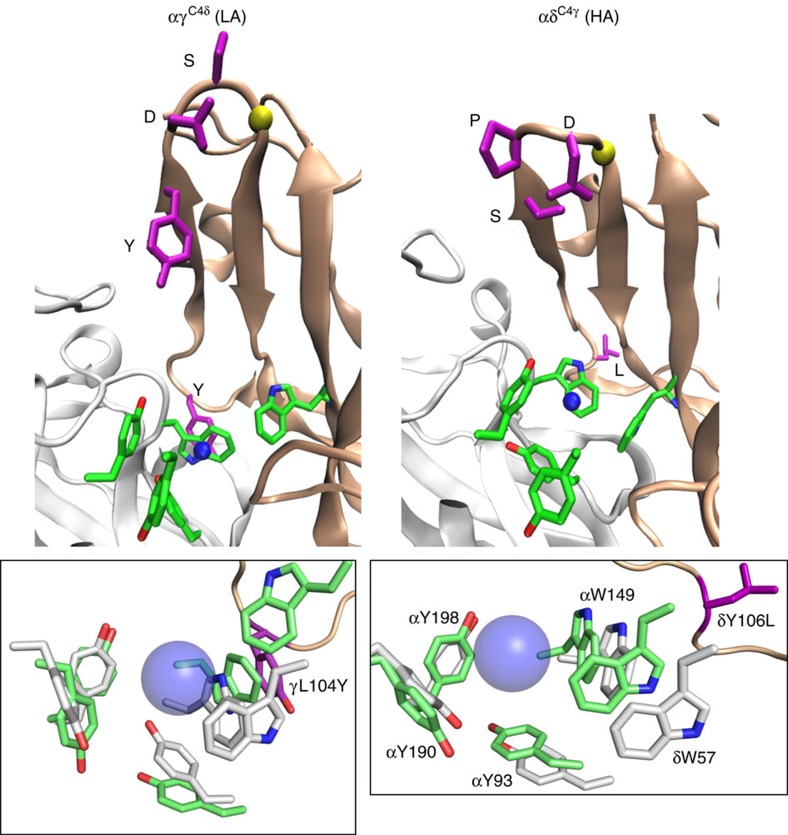
Representative snapshots of C4-swapped constructs. The snapshots have the lowest RMSD from the mean. Top: Low-resolution views; α-subunit, white; complementary subunit, tan; core aromatics, green; C4 amino acids, violet; ACh nitrogen, blue sphere; αC of conserved loop E glycine, yellow. Bottom: high-resolution view of core in the parent (white) and C4-swapped (green) constructs. In the higher affinity (HA) structures, the binding pocket is more compact and W55 is closer to agonist (quaternary ammonium group, large blue sphere).

**Figure 8:**
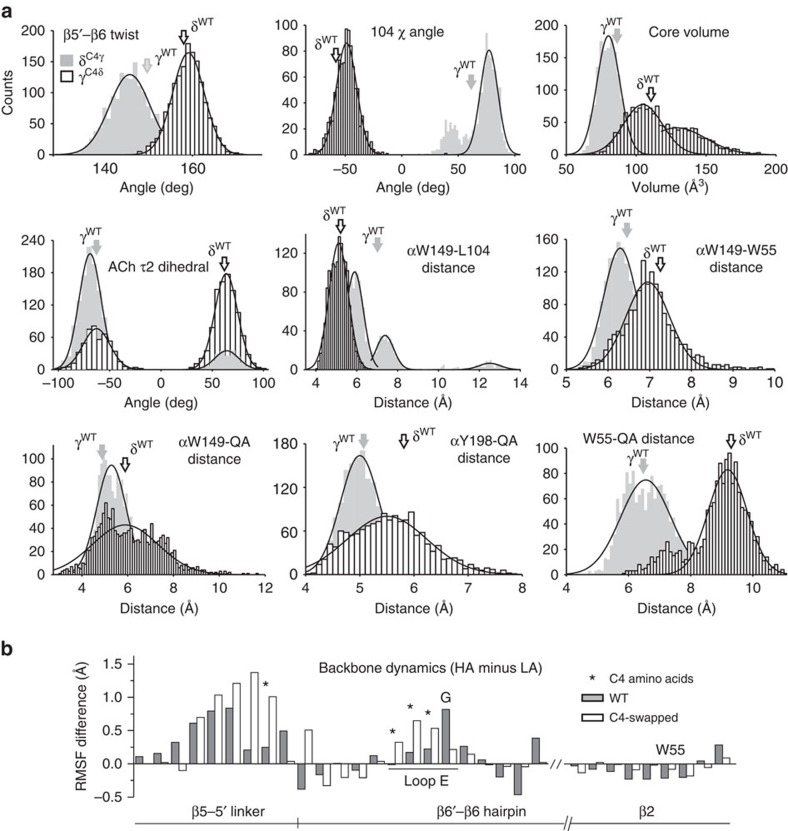
Quantitative analyses of MD results. (**a**) Structural parameters estimated from trajectories. Histograms are from C4-swaps; arrows are mean values from WT trajectories. (**b**) HA RMSF minus LA. WT, grey (αγ minus αδ) and C4-swap, white (αδ^C4γ^ minus αγ^C4δ^). The β5–β5′ linker and loop E are more flexible in HA structures, whereas the base of the hairpin and β2 near W55 are more flexible in LA structures (see Fig. 9c).

The two regions of interest were the core and the complementary β-sheet. Figure 7 shows representative snapshots from C4-swapped trajectories and Fig. 8a shows quantitative analyses of the HA versus LA structures. The structural parameters estimated from C4-swapped constructs were similar to those from the parent subunits of corresponding affinity.

**Figure 9:**
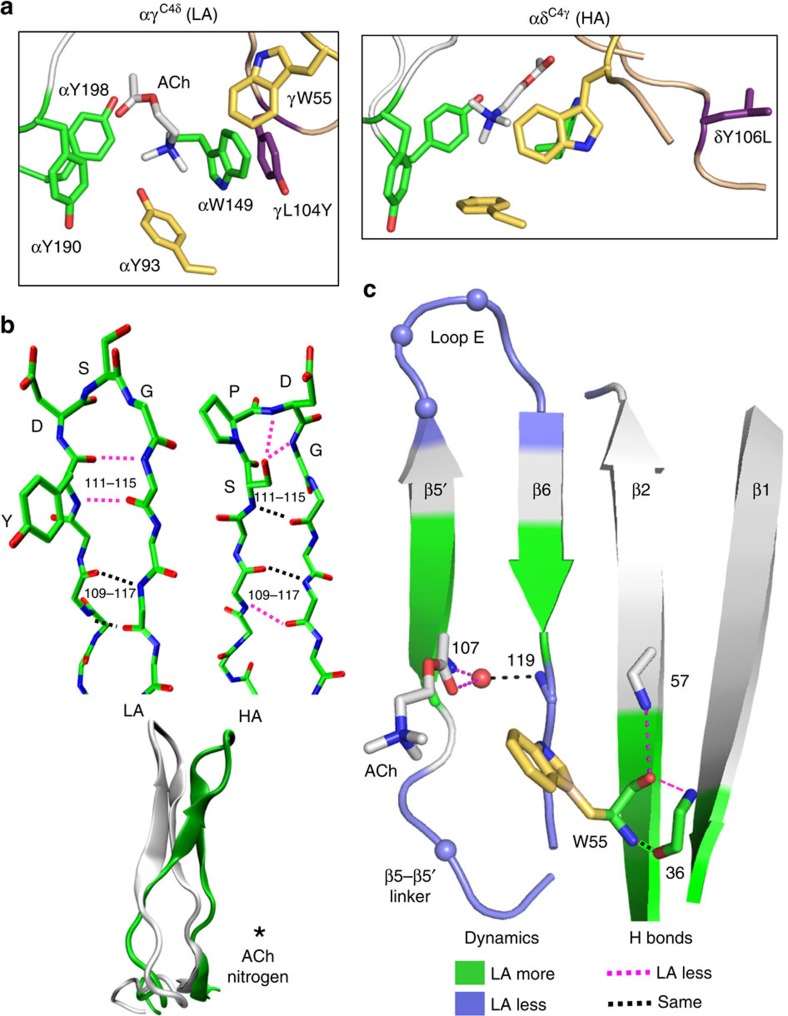
Structures suggest a mechanism for fetal versus adult affinity. (**a**) Representative snapshots of C4-swapped cores (LA, low affinity αδ^WT^/αγ^C4δ^ and HA, high affinity αγ^WT^/αδ^C4γ^). Left, the LA core is spread, γW55 is far from ACh, C4 residue γL104Y (purple) is face-on to αW149 and ACh is bent. Right, the HA core is compact, δW57-αW149-αY93 are orthogonal, δY106L points away from the agonist and ACh is straight. (**b**) H-bonds of the β5′–β6 hairpin. Top, dotted lines are H-bonds. pink, bonds that correlate with affinity (are ∼2 × more prevalent in simulations of LA or HA sites); black, bonds that are equally prevalent in LA and HA trajectories (Supplementary Table 2). Loop E (bold side-chain labels) is one residue shorter in LA structures. Bottom, in LA constructs the hairpin is straighter and less twisted. (**c**) Relative dynamics of the β-sheet backbone. LA is associated with a smaller RMSF (light blue) in the β5′ linker and loop E, and a greater RMSF (green) at the bottom half of the hairpin and regions surrounding W55. αC of the C4 amino acids, light blue spheres; structural water oxygen, red sphere; H-bonds, dashed lines; numbers are after γ-subunit. PDB accession number 3WIP; see Fig. 8 and Supplementary Table 2 for quantitative analyses.

Representative snapshots of the core from αγ^C4δ^ and αδ^C4γ^ trajectories are shown in Fig. 9a. In LA versus HA structures, the β5′ linker residue Y104 is close and face on with αW149, and β2 residue W55 is distant and not orthogonal to either αW149 or αY93. The structural differences of the α-subunit aromatic triad were less pronounced, but in the LA structures these side chains were all further from the agonist's quaternary amine (QA). Overall, the simulations indicate that the HA core is more compact, organized and stable.

In AChBP there is a structural water within the core that is H-bonded to ACh and the backbones of β5′ and β6^23^,^27^,^28^ (Fig. 1a). In the simulations, both this water and ACh were dynamic (Fig. 9c). The water-β6 H-bond had the same propensity in both HA and LA structures, but the H-bonds with ACh and β5 were less prevalent in LA trajectories (Supplementary Table 2). In the simulations, the N–C–C–O dihedral angle (τ2) of the neurotransmitter was bimodal, being either −60^o^ or +60^o^ (Figs 8a and 9a). The +60^o^ configuration was approximately five times more common in LA trajectories.

We also estimated structural parameters for the complementary surface, which is made up of the β5′ linker, the β5′–β6 hairpin (that includes loop E) and strand β2 (Fig. 1a). Figure 9b (top) shows inter-strand backbone H-bonds for the hairpin. In LA versus HA structures, loop E had three versus four residues, because with the HA C4 amino acids (SPD), the pre-proline backbone 111(O) is rotated out of the hairpin plane and fails to form an H-bond with the post-glycine backbone 115(N) (Supplementary Table 2). With LA amino acids (YDS), the 111–115 amide–oxygen H-bond is present so that the first residue is part of the hairpin rather than the loop. Also, in HA structures, the S111 side chain has an appropriate distance and geometry to H-bond with the loop E backbone (D113 or G114). The other difference in H-bonds is near the base of the hairpin, where the 109(N)–117(O) bond is less prevalent in LA structures. Figure 9b (bottom) shows that in the simulations, the overall hairpin is more upright (by ∼8^o^) and less twisted (by ∼9^o^; Fig. 8a) in LA structures.

There were fewer H-bonds between the W55 backbone and neighbouring residues in LA structures (Fig. 9c and Supplementary Table 2). Although the β2–β1 strand bond W55(N)–T36(O) was equally likely in LA and HA structures, the backbone H-bonds between W55(O)–T36(N) and W55(O)–E57(N) were less prevalent in LA.

Finally, we used root-mean-square fluctuation (RMSF) analyses to estimate the dynamics of the complementary β-sheet backbone (Fig. 8b). The results are summarized in Fig. 9c. In LA structures, the RMSF of the β5′ linker and loop E (that is, all C4 residues) was significantly smaller than in HA structures. This relative stability was accompanied by higher dynamics in other regions, in particular in the bottom half of the β hairpin and the β2 strand in the vicinity of W55. These differences in backbone dynamics correlate with the lower probability of H-bonds in LA structures at the base of the β-hairpin, with β5 and ACh and in β2 (Supplementary Table 2). In summary, in the simulations, lower affinity was associated with a straighter hairpin, a less-dynamic β5′ linker, a shorter and less-dynamic loop E, and a more-dynamic backbone near the core (base of hairpin, structural water and W55).

## Discussion

It was possible to swap *in vitro* the affinities of the fetal and adult agonist sites based on mutations identified *in silico*. Apparently, (i) the *Aplysia* AChBP is a good model of the endplate AChR resting αγ agonist site, (ii) classical force fields, short simulation times and approximate estimates of binding energy are adequate and (iii) other structural elements of the biological AChR (transmembrane and intracellular domains, lipid bilayer, post-translational modifications) are not major determinants of ACh affinity. The results suggest that simulations that are not state-of-the-art can nonetheless be used effectively as an engineering tool.

The C4 set of residues determines some of the functional differences between fetal versus adult AChRs. Swapping just these four side chains exchanges fetal versus adult AChR affinities and open-channel lifetimes. This suggests that the complementary, super-secondary β-sheet can adopt either a fetal-type (HA) or an adult-type (LA) conformation. The C4 mutation set does not, however, account for fetal versus adult differences in the unliganded gating equilibrium constant, gating-voltage dependence, single-channel conductance or ion selectivity. Nonetheless, it is now possible to control by C4 mutation(s) an AChR's affinity for the neurotransmitter and, by other mutations the allosteric constant, open-channel lifetime and, to some extent, single-channel conductance. This ability may lead to a better understanding of the reasons for the requirement of the γ-subunit in synaptogenesis.

The ability of the C4 mutation to swap open-channel lifetime was not expected because in adult AChRs most α-subunit mutations near the agonist sites have little or no influence on the open channel life time^29^. The slope of a log–log plot of opening rate versus gating equilibrium constant for a series of point mutations (*φ*) informs of whether the substitutions change the closed versus open lifetime, on a scale from 1 to 0 (refs 30, 31, 32). From the two sets of gating rate constants in the bimodal construct αγ^C4δ^ (Fig. 3b), we estimate that *φ*=0.57, a value that is characteristic of many residues in the α-subunit transmembrane domain^33^. We do not know whether this low *φ*-value can be attributed in general to complementary-side residues or to a difference between γ- and ɛ-subunits. An in-depth exploration of amino acid *φ*-values on the complementary surface and in the αγ core will be taken up, elsewhere.

The C4 mutation set does not account completely for differences in fetal versus adult AChR affinity. First, simulations showed a stable, high-to-low affinity exchange in αγ^C4δ^ but in electrophysiology experiments, the behaviour of this construct was bimodal (Fig. 3b). Apparently, with this swap, the energy barrier separating the alternative, complementary β-sheet folds was low enough to allow a reversible isomerization on the ∼20 ms time scale, which is far beyond the simulation time frame. Second, the effect of C4 swaps was only partial with regard to the affinity of W55A (both *in silico* and *in vitro*; Fig. 6). Third, the affinities of the C4 constructs for the partial agonists choline and TMA were not completely swapped (Supplementary Table 1). These results suggest that the C4 group does not fully account for all differences in fetal versus adult binding properties. The addition of mutations to C4, either from the original C10 set or elsewhere in the extracellular domain, might improve the fetal versus adult match in function. Also, some of the differences between simulated and experimental results may arise from long-range interactions that were not modelled.

Although the C4 combination was sufficient to exchange affinities, we cannot be sure that this set of substitutions is unique in this regard. There are 1,023 possible mutant combinations for the starting set of 10, of which we examined only 84 *in silico*. It is possible that other combinations could also generate the affinity swap. Also, the original selection criteria could have left out important residues from the starting set. For example, γE57/ɛG57/δD59 in the complementary β2 strand were not included in the starting set because the ɛ- and δ-side-chains are not homologous. Nonetheless, the ability of the C4 combination to swap affinity and lifetime suggests that the C4 residues are an important basis for the functional differences between fetal versus adult endplate AChRs.

Recently, it was found that in α4β2 neuronal nicotinic AChRs, three complementary-subunit residues determine differential agonist potency of α4–α4 versus α4–β2 binding sites^34^. The corresponding positions in the endplate AChR γ-subunit are L109, Y117 (in C10) and L119, none of which belong to the affinity-changing C4 group. This lack of correspondence may reflect a difference between these neuronal versus endplate AChRs, or may be traced to the benchmark of potency versus affinity. Nonetheless, the approach of combining simple and rapid simulations with *in vitro* energy estimates could be useful, in general, for revealing affinity-influencing amino acids in zones surrounding the core of ligand-binding sites in other ligand-gated ion channels.

In the simulations, the C4 mutations caused changes in structure that paralleled those that distinguished LA, αδ^WT^ and HA, αγ^WT^ sites (Fig. 8a). One of the C4 residues is in the β5′ linker, adjacent to the core, and the other three are in loop E and far from the core. Hence, the effects of the C4 side chains appear to take place by both local and non-local mechanisms, to generate a core that is more compact in HA versus LA structures (Fig. 9a).

The primary features that distinguish the complementary subunit in LA versus HA structures were as follows. (i) The β5′ linker is less dynamic, with the aromatic Y104 side chain close and face-on to αW149 and suggestive of a direct, π−π interaction. (ii) Loop E is one residue shorter. (iii) There are fewer H-bonds near the core, at the base of the hairpin with ACh and near W55 in β2. (iv) The W55 side chain is displaced from the core and not orthogonal to αW149 or αY93. (v) The loop E backbone is less dynamic and the β2 backbone near W55 is more dynamic. Overall, these differences suggest that three processes are required for C4 residues to generate a low affinity—unsettling the core by a local effect of the linker (104) side chain with αW149, loosening the β-sheet near the core by reducing the probability of H-bonds, and increasing the dynamics of the W55 backbone to unlock the special pair from the core.

We hypothesize that the dynamics and orientation of 104 are both determined by the aromatic versus aliphatic character of the side chain, which in LA is Y and in HA is L. The apparent π-stacking of the δY104 and αW149 aromatic rings (Fig. 9a, left) likely affects core architecture directly. This interaction may influence that between αW149 and ACh (∼0.7 kcal M^−1^ less favourable at adult sites^15^) and could also be a reason for the lower dynamics of the β5′ linker. The unsettling of the core by the linker Y is, however, not sufficient to lower affinity completely because the effect of the point, L↔Y mutation is relatively small (Fig. 5d), perhaps because γW55 remains locked in place. Recently, we showed that the αδ^C4γ^ mutations reduce modal changes in affinity induced by loop C mutations at the αδ site^35^. It is possible that increased compactness of the site, along with the loss of a π−π interaction with αW149 stabilizes the aromatic triad and affinity.

In LA constructs the β5′–β6 hairpin is straighter, and loop E is shorter and less dynamic. It is possible that these differences affect interactions between core residues and the agonist directly, but more likely they influence affinity indirectly by changing the H-bond network of the β-sheet to increase backbone dynamics near the core. The simulations suggest that in LA structures, the bottom half of the β-hairpin and near W55, locations that have fewer H-bonds, are more dynamic (Fig. 9c). Our hypothesis is that the loop E C4 residues unlock indirectly the W55 backbone and allow this side chain to move away from the core, reducing its interaction with its partner αY93 and diminishing the contribution of the special pair towards affinity.

The simulations also showed differences in the ACh molecule between LA and HA constructs. In LA structures, the ACh τ2 dihedral angle is +60^o^ rather than −60^o^, perhaps because the H-bond with water is less probable as a consequence of the greater backbone dynamics nearby. However, the extent to which the differences in agonist orientation and H-bonding relate to fetal versus adult affinity is uncertain. The agonist sites show a similar affinity difference for TMA (Fig. 1c, bottom), but this ligand does not have a rotatable bond and is too distant from the water to form a H-bond. We suspect that the core water is a factor that sets the higher affinity for ACh versus TMA, but may not be critical in determining the relative affinity of fetal versus adult sites.

The results suggest that both the structure and dynamics of complementary β-sheet are influenced by the C4 amino acids as a group, and that these residues are the bases, in part, for adult versus fetal endplate receptor function. At the agonist-site core, the orientation of the 104 side chain and dynamics of the W55 backbone together appear to generate the ∼30-fold affinity difference for the neurotransmitter. The simulations suggest that alternative conformations of the complementary β-sheet influence the architecture and affinity of the core. Further analyses of loop E interactions with loops A, B and C in the α-subunit (that hold other core aromatics) and with nearby residues in β2–β3 linker (the MIR) may further illuminate affinity mechanisms in AChRs.

## Methods

### Electrophysiology

AChRs were expressed in HEK cells by transient transfection of mouse endplate AChR subunits. Single-channel currents were recorded in the cell-attached patch configuration (23 °C). The bath solution was (mM): 142 KCl, 5.4 NaCl, 1.8 CaCl_2_, 1.7 MgCl_2_, 10 HEPES/KOH, pH 7.4 and the pipette solution was: 137 NaCl, 0.9 CaCl_2_, 2.7 KCl, 1.5 KH_2_PO_4_, 0.5 MgCl_2_ and 8.1 Na_2_HPO_4_, pH 7.3. To estimate the fully liganded gating equilibrium constant, a saturating concentration of agonist (100 mM; >100 × *K*_d_) was added to the pipette. The agonists were acetylcholine (ACh), tetrametylammonium (TMA), carbamylcholine (CCh) and choline. To place the gating rate constants into a range suitable for kinetic analysis, we added background mutations that changed the allosteric constant or changed the membrane voltage. Neither of these perturbations had any effect on affinity^36^,^37^.

### Experimental estimates of affinity and binding energy

Resting-state binding energy (in kcal M^−1^) is equal to +0.59 ln*K*_d_ (23 °C). *K*_d_ was estimated from single-channel current interval durations by using QUB software^38^. In one approach (Fig. 3a), *K*_d_ was estimated from the ratio of dissociation/association rate constants, which in turn were estimated by fitting interval durations across multiple [agonist] using a A+C↔AC↔AO scheme (one-site AChRs; A is the agonist, C is closed and O is open). In another approach (Figs 4 and 5d; Supplementary Table 1), *K*_d_ was estimated from a ratio of gating equilibrium constants, as follows. In both WT and mutant AChRs, *K*_d_≈*E*_0_/*E*_1_, where *E* is the C↔O gating equilibrium constant and the subscript indicates the number of bounds agonist (*E*_0_ is the allosteric constant)^39^. *E*_1_ was estimated as the gating equilibrium constant for a single site at high (agonist; full saturation, >5 × *K*_d_). *E*_0_ was estimated from the gating equilibrium constant in the absence of any agonists using a constitutively active background^40^,^41^. In the cross-concentration approach, the membrane was held at −100 mV, and in experiments at high (agonist) the membrane was depolarized to +70 mV to reduce channel block by the agonist. Membrane potential has no effect on *K*_d_ (ref. 37). When both approaches were used for the same construct, the *K*_d_ estimates agreed.

### Protein engineering

The mutations were incorporated into AChR subunits using the QuickChange site-directed mutagenesis kit (Agilent Technologies, CA) and were verified by nucleotide sequencing. Many AChR mutations away from the agonist sites only influence *E*_0_ (ref. 33), which is 7.4 or 0.52 × 10^−7^ in WT adult or fetal AChRs at −100 mV (refs 40, 41). We measured *E*_0_ for every mutant construct by adding background mutations that increased it by known extents, but had no effect on affinity^36^. In selecting the backgrounds, we chose those that were energetically independent, so that the aggregate *E*_0_ was the product of the WT value and the individual fold changes. The values shown in Fig. 5d have been corrected for the backgrounds.

To study AChRs having just one functional binding site, we added mutations to the ɛ-, γ-, δ-subunits that eliminate binding at the mutated site^24^. To make αγ- or αɛ-only AChRs, we added δP123R, and to make αδ-only, we added ɛ/γP121R, sometimes in combination with γW55R. These mutations also change *E*_0_, which was measured for each knock-out construct.

### Homology model

**Figure 10:**
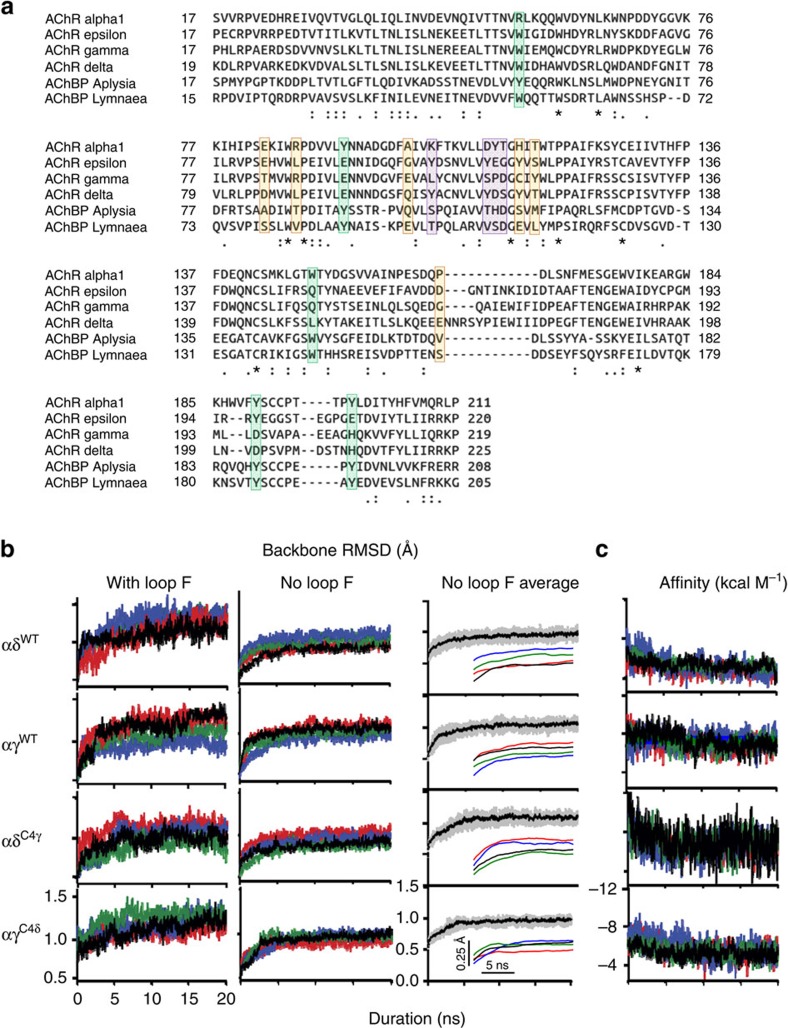
Sequence alignment and stability of models. (**a**) Sequence alignment of the extracellular domain of AChR subunits (mouse) and AChBPs. Highlighted residues are: core aromatics, green; C4, violet; other members of C10, orange. (**b**) Temporal evolution of simulated root-mean-square-deviation (RMSD) in WT and C4-swapped constructs. Left, total RMSD; each colour is a different trajectory. Centre, RMSD after removing a section of loop F. Right, average (black) + s.d. (grey) of all four trajectories (loop F removed; inset is rolling 5 ns average of each trajectory). All three plots show that the structure is stabilized by ∼10 ns. (**c**) Temporal evolution of simulated ACh binding energy (loop F included).

The homology model of the extracellular domain of the fetal-type AChR was built based on the *Aplysia californica* ACh binding protein (AChBP) bound to epibatidine (pdb ID: 2BYQ) by using MODELLER^42^. The sequences of AChBP and AChR subunits were aligned using CLUSTALX^43^ (Fig. 10a). The AChR subunits share ∼20% sequence identity with *Aplysia* AChBP. AChR subunits were modelled simultaneously so that spatial reciprocity was maintained at the interfaces. Residues 128 and 142 in the α-subunit and the corresponding residues in the other subunits were constrained as disulfide bonds. A protocol of conjugate gradient optimization, simulated annealing and molecular dynamics were used to refine the structure.

First, 100 structural models were generated. MODELLER has various assessment methods and objective functions to test the validity of a homology model (such as molpdf and DOPE scores), but these are not recommended to be used for multi-chain proteins^44^. Therefore, we used PROCHECK scores based on G-factor^45^ to rank the models. The model with the best G-factor score was chosen for docking and simulations^46^. The top five models based on G-factor were similar in both structure (backbone root-mean-square deviation (RMSD) <0.7 Å; the equilibrated structure RMSD from the simulations was 1.2 Å) and scores. The selected model also ranked high in the MODELLER scores (first in molpdf and fifth in DOPE score), bad contacts (third) and Ramachandran criteria (third). Further minimization of the selected model reduced the bad contacts to zero.

### Ligand docking

ACh and TMA were docked at the αδ and αγ binding sites using the Lamarckian genetic algorithm in AUTODOCK^47^. ACh and the core aromatic residues were allowed to be flexible, and 30 Å cubic search grid was used at the expected binding site with 0.375 Å grid spacing. Docked structures were analysed and selected on the basis of lowest energy and RMSD clustering. The CHARMM force field parameters for ACh and TMA were obtained from the Charmm Generalized Small Molecule Force Field webserver (CGenFF)^48^,^49^.

### MD simulations

Point mutations were introduced *in silico* using the VMD *mutator* plug-in^50^. AChRs with ligands or mutations were optimized and equilibrated by using energy minimization and MD simulation. The system was solvated in a water box with TIP3P water model^51^ and the box boundary was extended at least to 10 Å from the periphery of the protein in each dimension. Na^+^ and Cl^−^ ions were added to neutralize the system and bring it to an ionic concentration of 150 mM NaCl.

Molecular dynamics simulations were run using NAMD^52^ version 2.8, with the CHARMM27 force field^53^. First, a 20,000-step minimization was done using the steepest descent method, and with gradual release of restraints on the protein backbone. The system was heated to 300 K over 100 ps, a 500 ps equilibration run was performed in the NVT ensemble and then 20 ns MD simulations were performed in the NPT ensemble at a temperature of 300 K and pressure of 1 atm using the Nosé–Hoover method^54^. Following minimization, harmonic constraints (force constant=1 kcal M^−1^ Å^−2^) were applied on the Cα atoms of residues, which were >25 Å away from the ligand. These restraints maintained the global backbone conformation of the model while allowing relaxation of all side chains.

Periodic boundary conditions were applied. A 10 Å switching distance and a 12 Å cutoff distance were used for non-bonded interactions. The particle mesh Ewald method^55^ was used to calculate long-range electrostatic interactions. The SHAKE algorithm^56^ was used to constrain bond lengths of hydrogen-containing bonds, which allows a time step of 2 fs for MD simulations. Four MD simulation trajectories were obtained for each system. The coordinates of the systems were saved every 1 ps during MD simulations for later analyses. The protein RMSD became stable by the first 10 ns (Fig. 10b). All the analyses and binding energy calculations were done on snapshots extracted every 20 ps over the last 10 ns of each trajectory. The ensemble for each system therefore contained 2,000 snapshots of the system.

### Calculation of affinity

A total ligand-protein binding energy (Δ*E*) was estimated as described elsewhere^57^. In brief, this energy was calculated using a continuum solvent model from an ensemble of 2,000 snapshots:


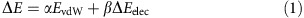


where *E*_vdW_ is the van der Waals contribution and Δ*E*_elec_ and is the electrostatic contribution calculated using the Poisson−Boltzmann (PB) method (PBEQ module of CHARMM^58^ in a salt concentration of 140 mM). The molecular dielectric surface was defined by a probe radius of 1.4 Å. Previously optimized atomic Born radii^59^ for the 20 amino acids were used to estimate the electrostatic free energy in explicit water molecules. The dielectric constant of the protein interior and the aqueous environment were set to 4 and 80, respectively.

The coefficients *α* and *β* in equation (1) were evaluated by comparing simulated and experimental Δ*E* values for alanine mutations and optimizing the coefficients to reproduce the experimental energies (Fig. 1b). We calculated *in silico* binding energies for the WT (with ACh/TMA) and Ala mutations at each of the core aromatic residues (α93, α149, α190, α198 and δ55), as described above. Values of *α* and *β* were scanned within a range of 0–2 to minimize the RMSE (root-mean-square error) between simulated and experimental energies for total 13 sample points:


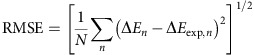


where *n* is each sample point and *N*=13. The overall fitting quality was tested by the coefficient of multiple determination *R*^2^. The result was *α*=0.22 and *β*=1.24. The *α* and *β* values for barnase–barstar (0.17 and 1 (ref. 57)) were shown previously to reproduce the fetal versus adult ACh binding energy difference^15^. The small *α* is possibly due to the loss of protein–water VdW contacts in the AChR, whereas the larger *β* (>1) may be due to cation–π interactions between the quaternary ammonium group of the ligand and the binding site aromatics, which is unaccounted for by the classical force field. With these values of *α* and *β*, the RMSE between simulated and experimental results were at a minimum (0.34 kcal M^−1^; *R*^2^=0.92), with a correlation coefficient of 0.97. To test the generality of these parameters, we plotted simulated and experimental affinities for two additional, non-alanine mutations (δY106L and γL104Y) in Fig. 1b. These fell on the same regression line.

The approximate binding energy calculated from MD simulations using the above method (Δ*E*) and the free energy estimated from single-channel currents (+0.59 ln(*K*_d_)) are not equivalent. However, the entropy contribution to ligand binding energy has been experimentally shown to be negligible in case of muscle-type AChRs^22^, and here we show that changes in these quantities can be compared for the purpose of engineering biological AChRs. In the text, we use ‘affinity' for both the *in silico* and *in vitro* binding energy estimates.

### Structure analyses

The geometric centres of the aromatic rings of interest and the ACh quaternary amine (QA) nitrogen were used as reference points. Structural analyses were done using the last 10 ns of each trajectory for the hetro-pentamer simulations. We measured all the distances and angles using VMD.

The volume of the ligand binding pocket was calculated by joining the centroids of the aromatic rings to form two adjoining tetrahedrons. The volume of each tetrahedron was calculated using the three-simplex determinant method from the coordinates of the vertices, as described elsewhere^15^. The β-hairpin twist was measured from the angle between the central axial vectors of β5' and β6. The central axis was defined by the least squares linear regression fit of the coordinates of the backbone atoms of residues 107–110 in β5' and residues 115–118 in β6 (γ-subunit numbers). The regression fit was calculated by singular value decomposition of the coordinates.

To identify a hydrogen bond between two atoms (that is, acceptor and donor) a donor–acceptor distance of <3.5 Å and a H-donor–acceptor angle of ≤30° were used as criteria^60^. We used VMD to identify and calculate the occupancies of all H-bonds within the last 10-ns ensemble.

### RMSD and RMSF analyses

To assess the conformational stability of the MD simulations, we calculated the RMSD of all backbone atoms in the α- plus complementary subunit relative to the starting structures. The RMSD plots showed a small amount of drift over time in some of the trajectories (Fig. 10b, left). We determined that most of this drift came from the complementary-side loop F, which was highly flexible and disorganized. We repeated the RMSD analyses after removing a part of loop F from the calculation (residues 166–183 in γ and 164–177 in δ), and both the drift and variance in the RMSD were reduced significantly (Fig. 10b, centre). The plots show that the system stabilized within ∼10 ns. This region of loop F had been modelled *ab-initio* into the homology model because there is no corresponding region in AChBP. To test the role of loop F in affinity, we partitioned the binding energy (Equation 1) into contributions from individual residues using CHARMM. None of the loop F residues that were excluded from the RMSD calculations were in the top 5% (*n*∼25) of those contributing to affinity, that comprise ∼98% of the total binding energy. The choice of 5% corresponds to a *P* value of 0.05.

As a further test of stability, we calculated a running average on the RMSD (without loop F) using a rolling window of 5 ns (Fig. 10b, right inset). The slopes of the regression lines fitted to the last 10 ns were small. The overall mean absolute slope value was 0.002+0.001 Å ns^−1^ (+s.d) for all the four constructs combined (*n*=16 trajectories). In addition, we calculated the average RMSD for the four trajectories for each construct (Fig. 10b, right; s.d. calculated from 5-ns bins). The drifts in these averages during the last 10 ns (∼0.02 Å) were smaller than the s.d.s. of the fluctuations (∼0.07 Å). These tests establish that all of the systems became stable within 10 ns and could be used for affinity estimation.

To compare the flexibility of ligand binding interface between the two states, we performed RMSF analysis on the backbone atoms based on the last 10 ns of the MD simulations. The RMSF of each residue was calculated with respect to the average structure of the ensemble using VMD version-1.9. This gives a residue-wise average of all possible fluctuations in the trajectories.

## Additional information

**How to cite this article:** Nayak, T. K. *et al.* Structural correlates of affinity in fetal versus adult endplate nicotinic receptors. *Nat. Commun.* 7:11352 doi: 10.1038/ncomms11352 (2016).

## Supplementary Material

Supplementary Information Supplementary Figure 1 and Supplementary Tables 1-2
