# Supplementary material for: Structural correlates of affinity in fetal versus adult endplate nicotinic receptors
Source: Nat Commun. 2016 Apr 22;7:11352. doi: 10.1038/ncomms11352 (PMC4845029; doi:10.1038/ncomms11352)
Supplement: Supplementary Information — Supplementary Figure 1 and Supplementary Tables 1-2 [file ncomms11352-s1.pdf]

Supplementary Figure 1

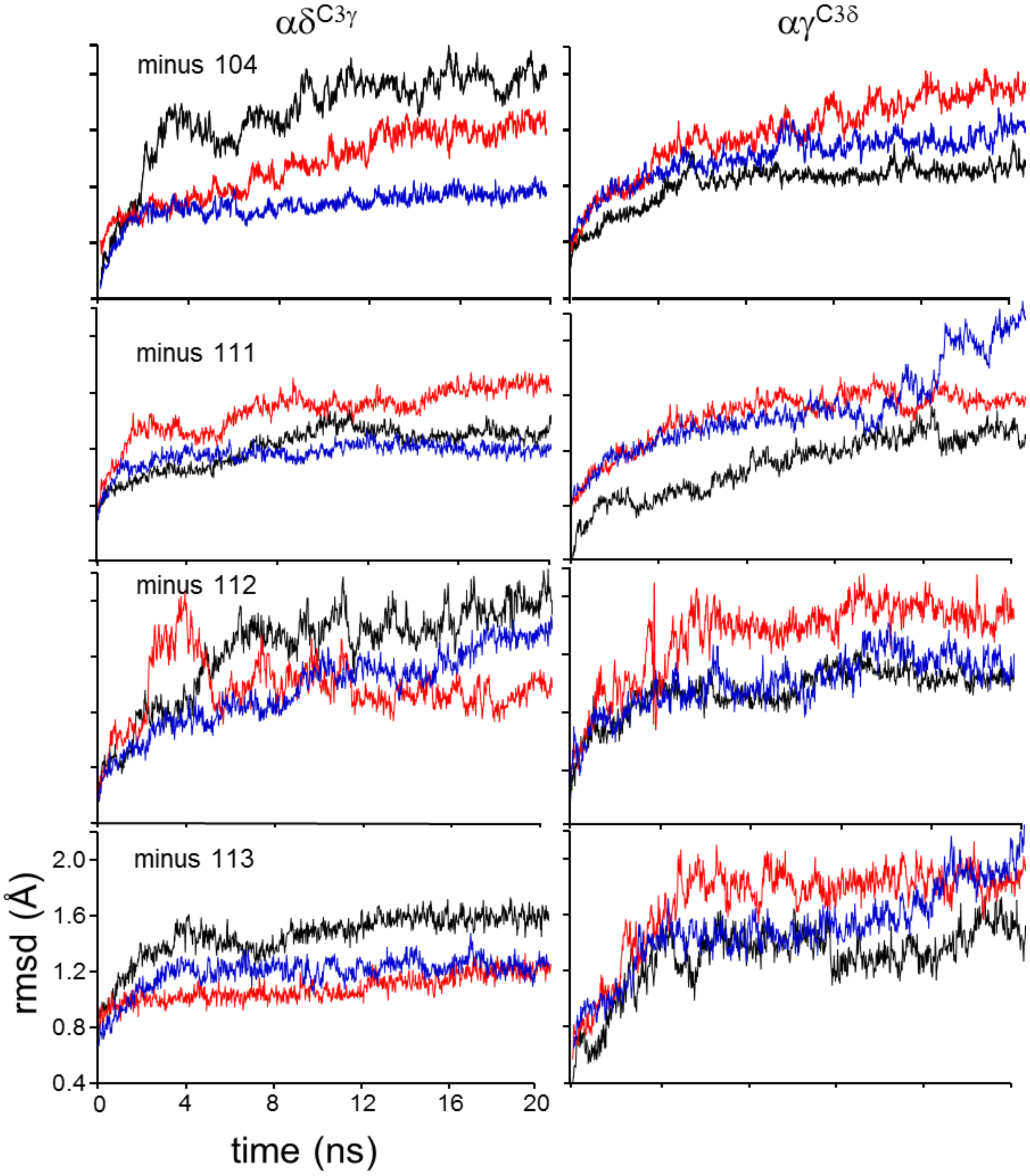

Temporal evolution of simulated root-mean-square-deviation (RMSD) in C3-swapped constructs (panels: C4 construct minus 1 residue each). Left, $\alpha\delta^{C3\gamma}$ RMSDs and right, $\alpha\gamma^{C3\delta}$ RMSDs; every color is a different trajectory. Trajectories of these 3-mutant swaps were unstable with fluctuating and divergent RMSD.

Supplementary Table 1

In vitro affinities from electrophysiology experiments

WT→C4	agonist	K_d (μM)		free energy (kcal·M^{-1})		% success
		WT	swapped	WT	swapped	
αδ→αγ	ACh	125	3.6	-5.3	-7.4	116
	TMA	810	45	-4.2	-5.9	88
αε→αγ	ACh	175	7.0	-5.1	-7.0	93
	TMA	1350	175	-3.9	-5.1	54
	Cho	10300	810	-2.7	-4.2	83
αγ→αδ	ACh*	6.0	75	-7.1	-5.6	83
	TMA	30	350	-6.2	-4.7	76
	CCh	3.0	45	-7.5	-5.9	64
αγ→αε	ACh	6.0	90	-7.1	-5.5	80
	TMA	30	250	-6.2	-4.9	56
	Cho	490	2650	-4.5	-3.5	56

Free energy (kcal·M^{-1})=+0.59lnK_d (in M). % success is with regard to energy and is equal to [(swapped-WT)/(WT^{target}-WT)]×100, where WT^{target} is the energy of the C4-intended site (for example, αγ-site energy in $\alpha\delta^{C4\gamma}$). *, estimated from the low P_O cluster population (see Fig. 3b).

Supplementary Table 2

H-bonds

Atoms (γ subunit)	$\alpha\delta^{WT}$(LA)	$\alpha\gamma^{WT}$(HA)
L109 (N) - Y117 (O)	0.5	1.0
L109 (O) - Y117 (N)	0.8	0.8
S111 (N) - C115 (O)	1.0	0.5
S111 (O) - C115 (N)	0.4	0.0
S111 (O) – G114 (N)	0.3	0.0
S111 (OG) - G114 (N)	-	0.9
S111 (OG) - D113 (N)	-	0.8
W55 (O) - E57 (N)	0.0	0.4
T36 (N) - W55 (O)	0.1	0.5
T36 (O) - W55 (N)	1.0	0.9
N107 (N) - O (water)	0.5	1.0
L119 (N) - O (water)	0.8	0.7
ACh (O) - O (water)	0.3	0.7

Fraction of trajectories in which each H-bond is present.
